# Genetic variance in human disease – modelling the future of genomic medicine

**DOI:** 10.1242/dmm.049700

**Published:** 2022-06-30

**Authors:** Monkol Lek, Julija Hmeljak, Kirsty M. Hooper

**Affiliations:** 1 Department of Genetics, Yale University School of Medicine, New Haven, CT 06510, USA; 2 The Company of Biologists, Bidder Building, Station Road, Cambridge CB24 9LF, UK

## Abstract

**Summary:** DMM is launching a new Subject Focus on genetic variance in human disease. Here, we discuss this ongoing series of invited articles and reflect on advances in understanding the genotype–phenotype complexities in disease.

2021 marked the 20th anniversary of the publication of the draft human reference genome. Since then, many technological advances have been made, and the wealth of human genomic and transcriptomic data has been expanded, deepened and diversified (Lek and Mardis, 2021). 2022 was another landmark year for the genetic research community, with the completion of the entire telomere-to-telomere reference human genome assembly (Church, 2022; Nurk et al., 2022). These developments strive towards a deeper understanding of variation in the human genome that impacts both health and disease, but genomics cannot achieve this alone. We must be conscious of how we interpret these data and integrate them with phenotypes to develop a holistic view of human disease, its causes and consequences, across the global human population. With this in mind, researchers continue to develop advanced analytical tools to assess human genetic variation and a spectrum of model systems to understand its phenotypic consequences in human disease. In this issue of Disease Models & Mechanisms, we launch a new Subject Focus ‘Genetic variance in human disease: decoding diversity to advance modern medicine’, spearheaded by Associate Editor Monkol Lek and Editor-in-Chief Liz Patton. This ongoing series of invited articles reflects on the latest advances in our understanding of genetic variance and genotype–phenotype complexities in disease. New Interview, Perspective and Review articles will discuss model systems and tools, as well as striking examples of how combining human genomic data with experimental systems helps us understand the broad spectrum of disease presentation, assess risk and identify treatment opportunities.

**Figure:**
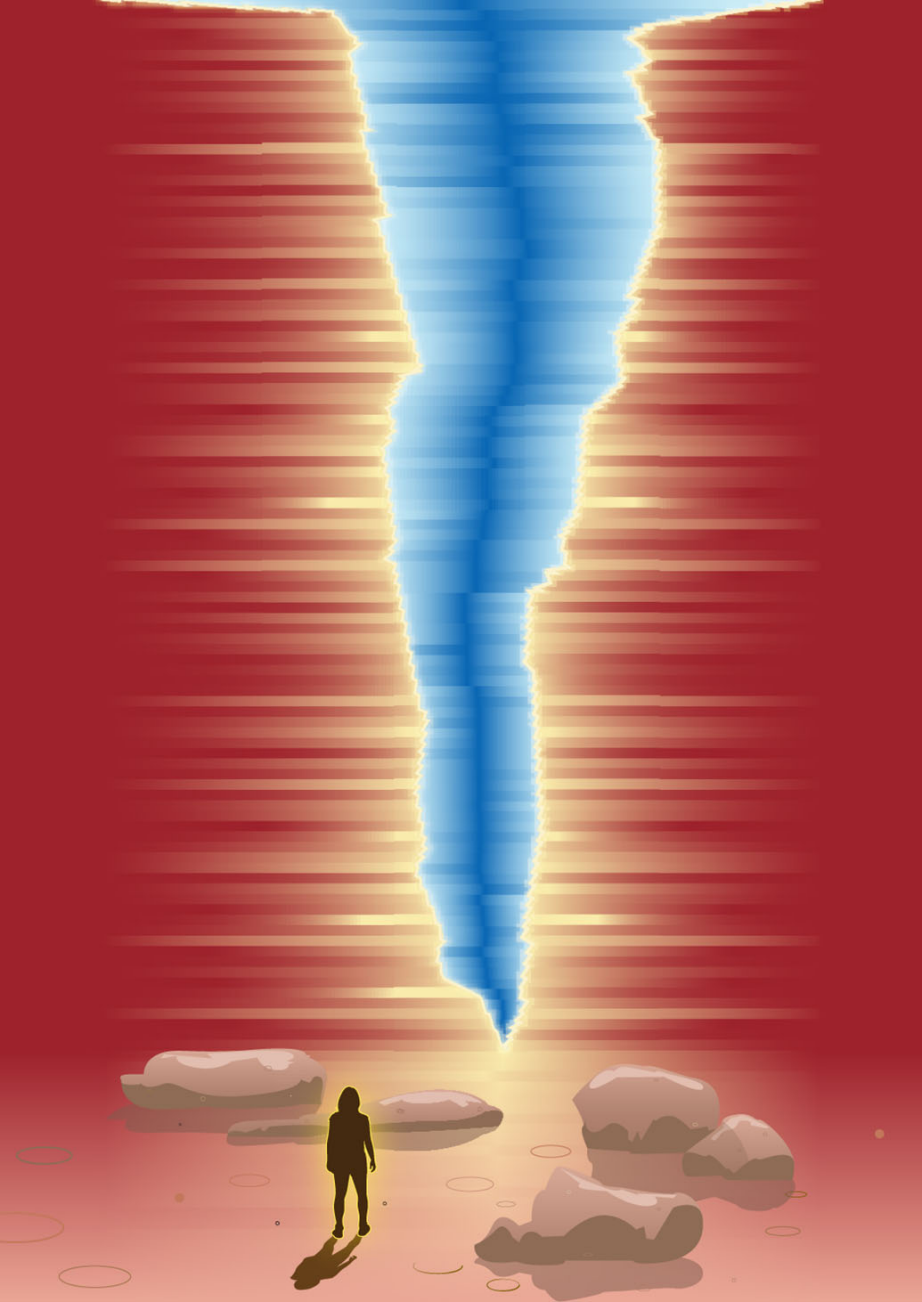

As in-depth human genome data expanded, we increased the pool of variants of uncertain (clinical) significance (Richards et al., 2015), which poses an immense challenge for translating genotype to phenotype in the context of human disease. When a novel variant is associated with a disease, it is important to examine the associated phenotype in suitable model systems, whether it be *in silico*, *in vitro* or *in vivo*. Advances in multi-omics and imaging alongside improved integration across model systems allows thorough examination of disease phenotypes and the degree to which genetic variance affects them. Furthermore, rapid screening and in-depth phenotyping of potential disease-causing variants in organisms such as *Drosophila* (Wall et al., 2021), zebrafish (Isiaku et al., 2021), *Caenorhabditis elegans* (Lange et al., 2021) and yeast (Young et al., 2020) facilitate mechanistic discovery and translation. For instance, humanised yeast can be used to rapidly assess vast numbers of variants within a single gene to predict pathogenicity (Kachroo et al., 2022). Similar screens can also be achieved in cell lines or *in silico* to support computational variant effect predictors (Livesey and Marsh, 2022). Furthermore, expansive biobanks and improved access to electronic health records can augment phenotyping efforts with valuable human data (Schalkamp et al., 2022; Lek, 2022). To effectively translate this wide range of genotypic and phenotypic data, investigators need the support of sophisticated analysis tools, integrated databases and centralised reagent-sharing networks (Bellen, 2022).

In rare and ultra-rare disease, low numbers of patients can make it difficult to pinpoint a genetic diagnosis and even harder to develop effective treatments. A solution to this urgent issue is enhancing global collaboration and data sharing, enabled via platforms such as the Matchmaker Exchange (Philippakis et al., 2015), helping both to solve the diagnostic odyssey for rare disease patients and to advance treatments (Kropp et al., 2021; Lek, 2022). By contrast, some disease-associated variants can be very common, such as the alcohol flushing syndrome-associated *ALDH2* variant. This single variant has been linked to a multitude of phenotypes, yet there is still more to explore beyond its alcohol-related effects (Chen et al., 2022). Whether a variant is rare or common, accurately and thoroughly assessing the phenotype remains a challenge, but with collaborative efforts, researchers can begin to connect the dots.

Another area of difficulty is the exploration of diseases with complex genetic aetiology. Advances in computational models that assess polygenic risk scores are necessary to carefully evaluate disease susceptibility (Lewis et al., 2021). Hand in hand with genetic variation analyses, heterogeneity in aetiology and phenotype can be untangled by deep phenotyping in patient cohorts. Computational analysis of rich clinical datasets can improve risk prediction, diagnosis, prognosis and treatment for many complex diseases, such as Parkinson's disease (Schalkamp et al., 2022). This, however, is another aspect of human disease research where expanding data in diverse populations is imperative. We need to understand the effect of both benign and pathogenic variants to truly decipher the genetics of disease, but to address this properly and effectively, this needs to be done across global populations (Lek and Mardis, 2021; Lek, 2022). As modern medicine heavily relies on genetics, we must ensure that these advances will benefit everyone (Olopade, 2021; Raghavan, 2022).

DMM's new Subject Focus ‘Genetic variance in human disease: decoding diversity to advance modern medicine’ will continue to highlight how researchers are fine-tuning genotypic and phenotypic data to infer disease biology, risk and therapy. Technological advances have proven vital to build rich datasets, but studies also need to consider genetic variation between populations to mitigate health disparities. As modern medicine embraces genomics, global collaborations and knowledge sharing should be a priority. By curating an ongoing series of invited articles that examine these nuanced and complex issues, we hope to support the community in building an inclusive future for translational genomic medicine.
